# G-Quadruplexes in Nuclear Biomolecular Condensates

**DOI:** 10.3390/genes14051076

**Published:** 2023-05-13

**Authors:** Iuliia Pavlova, Mikhail Iudin, Anastasiya Surdina, Vjacheslav Severov, Anna Varizhuk

**Affiliations:** 1 Lopukhin Federal Research and Clinical Center of Physical-Chemical Medicine, 119435 Moscow, Russia; 2 Moscow Institute of Physics and Technology, 141701 Dolgoprudny, Russia

**Keywords:** liquid–liquid phase separation, membraneless condensates, G-quadruplex, chromatin structure

## Abstract

G-quadruplexes (G4s) have long been implicated in the regulation of chromatin packaging and gene expression. These processes require or are accelerated by the separation of related proteins into liquid condensates on DNA/RNA matrices. While cytoplasmic G4s are acknowledged scaffolds of potentially pathogenic condensates, the possible contribution of G4s to phase transitions in the nucleus has only recently come to light. In this review, we summarize the growing evidence for the G4-dependent assembly of biomolecular condensates at telomeres and transcription initiation sites, as well as nucleoli, speckles, and paraspeckles. The limitations of the underlying assays and the remaining open questions are outlined. We also discuss the molecular basis for the apparent permissive role of G4s in the in vitro condensate assembly based on the interactome data. To highlight the prospects and risks of G4-targeting therapies with respect to the phase transitions, we also touch upon the reported effects of G4-stabilizing small molecules on nuclear biomolecular condensates.

## 1. Introduction

G-quadruplexes (G4s) are planar arrangements of Hoogsteen bonded guanine tetrads. They can be formed through the association of several nucleic acid strands that harbor G2+ tracts or an intramolecular tetrahelical folding of a single strand [1]. Within genomic DNA, intramolecular G4 folding is favored by a low nucleosome density and negative supercoiling [2,3]. Chromatin immunoprecipitation with the quadruplex-specific antibodies BG4 [4] and 1H6 [5] or an artificial protein probe PG4 [6] revealed the G4 association with DNA damage hotspots [7] and an abundance in the regulatory genomic regions, including promotors and 5′-UTRs [8,9], telomeres [10], and boundaries of topologically associating domains [11], etc. Such a distribution pointed to a G4 relevance for reparation, transcription, genome integrity maintenance, and chromatin remodeling, which has been analyzed comprehensively from the biological perspective [12,13,14,15,16,17]. Recently, these processes have been reconsidered from the biophysical perspective to account for their compartmentalization in biomacromolecular condensates, also known as membraneless organelles [18,19,20]. To complement the resulting updated view on chromatin packaging and gene expression control, we readdressed the G4s in the regulatory DNA regions and summarized the evidence for their contribution to condensate formation.

**Figure 1:**
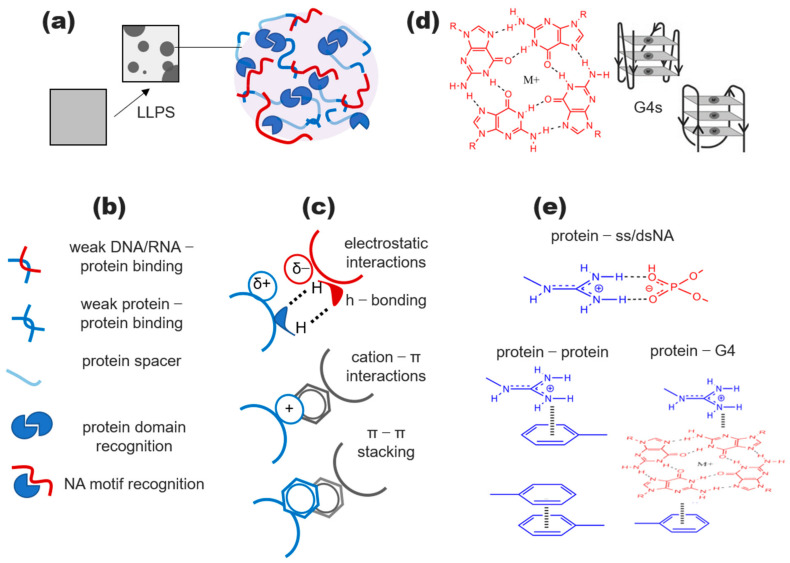
Molecular grammar of the biomolecular condensates and presumed G4 contacts within the condensates. (**a**) Schematic representation of the condensate formation through the liquid–liquid phase separation (LLPS). (**b**) Typical constituents and their interactions. (**c**) Major types of transient LLPS-driving contacts. (**d**) Schematic representation of the G4 structures. (**e**) Typical examples of the transient contacts in the protein mixtures with ss/dsNA and G4 NA.

Biomacromolecular condensates are assembled spontaneously through the liquid–liquid phase separation (LLPS) of biopolymers, typically nucleic acids and proteins with low-complexity domains (LCDs) or intrinsically disordered regions (IDRs) [18,21]. Such proteins and nucleic acids are prone to weak multivalent homo/heterotypical interactions (Figure 1), which outcompete the water–biopolymer interactions within a certain polymer concentration range, favoring solution demixing into polymer-depleted and condensed liquid phases [22,23,24,25]. The resulting increase in the local macromolecule concentration can promote the assembly of multicomponent complexes, facilitate the enzyme–substrate recognition, and increase the reaction rates [26]. At the same time, the LLPS-mediated compartmentalization enables the reversible isolation of excessive or toxic biopolymers from the bulk cellular media [27]. Assuming the G4s trigger or assist in the phase separation, G4-(de)stabilizing endogenous or exogenous ligands (potential drugs) may have profound effects on the proteostasis. This possibility should be taken into account in the development of G4-targeted therapeutics.

RNA G4s have recently emerged as prospective drivers for various phase transitions in the cytoplasm [28]. The evidence for their contribution to the assembly of stress granules and toxic aggregates associated with neurodegenerative diseases [29,30] has reinforced the interest in RNA G4 ligands as drug candidates. Major advances in this field have been discussed elsewhere [31,32]. In this review, we focus on the possible links between the G4s and LLPS in the nucleus. We present a brief analysis of the G4-binding proteins that readily form condensates in tube/in cell and focus on a particularly enriched functional category, namely the RNA interactors functioning in both the nuclei and the cytoplasm. Next, we discuss the major types of nuclear condensates that contain G4-prone DNA/RNA and comment on their involvement in the basic LLPS-driven cellular processes, such as transcription and DNA reparation. Much attention is paid to the presumed mechanisms of the biocondensate formation in the presence of G4 RNA/DNA. Finally, we outline the future prospects for investigating the G4-dependent LLPS and its modulation with G4-binding small molecules.

## 2. RNA-Binding LLPS Drivers Are Abundant in the G4 Interactome

A substantial number of acknowledged G4 interactors have been identified using non-high-throughput methods and assays that do not fully recapitulate the intracellular conditions [33]. Early semi-comprehensive studies of the G4 interactome relied on G4 profiling against human protein microarrays [34] or affinity purification of the G4 binders from nuclear extracts followed by their mass-spectrometry-based identification and quantitative analysis [35]. The functional analysis of the candidate G4 interactors obtained in those assays showed an enrichment in the RNA-binding proteins, transcription factors (TFs) or RNA processing factors, helicases, and chromatin remodelers [36,37]. Most of them are summarized in the G4 interacting protein database [38]. The recently developed alternative techniques, namely enhanced cross-linking and immunoprecipitation (eCLIP) and co-binding-mediated protein profiling (CBMPP), using a functionalized G4-recognizing small molecule probe that was cross-linked in situ with the G4-recognizing proteins to substantially expand the repertoire of the candidate G4 interactors [39,40]. In addition to the abovementioned overlapping functional categories, eCLIP and CBMPP revealed the prevalence of the DNA damage response (DDR)- and infection response-related proteins in the G4 interactome.

The established or predicted LLPS drivers can be found in each category of the G4 binders. Those related to infection sensing and innate immune signaling include the DEAD box helicases, such as DDX1 and DDX24 [39], DDX5 [41], Dbp1 [42], and Dbp2 [43]. These helicases shuttle between the nucleus, where they control transcription, and the cytoplasm, where they sense viral nucleic acids and contribute to the antiviral response, e.g., via promoting the assembly of stress granules through their LLPS-prone IDRs [44,45]. In addition to the G4 DNA, DEAD box helicases recognize RNA in a sequence-dependent manner. They are regarded as major ATP-dependent regulators of all the RNA-containing biomolecular condensates [46,47].

The other G4-recognizing LLPS drivers from the RNA binding category include the plasminogen activator inhibitor 1 mRNA binding protein (SERBP1) [48], nucleophosmin [49], nucleolin (NCL) [50], heterogenous ribonucleoprotein A1 (hnRNP A1) [51,52], and fused in sarcoma (FUS) [53,54].

**Figure 2:**
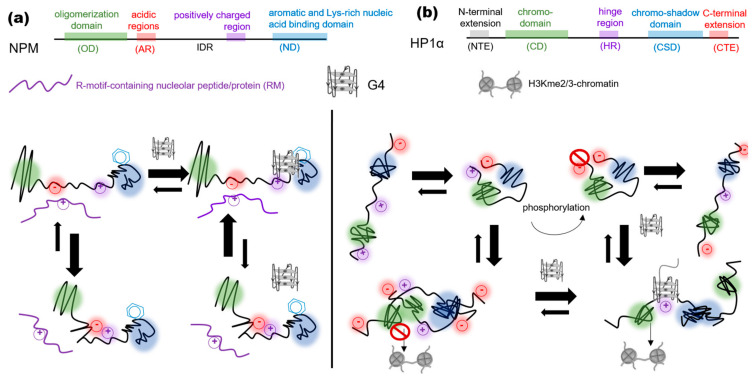
Effects of the G4s on the conformational transitions of the LLPS-driving proteins. (**a**) Nucleophosmin (NPM). The IDR- and ND-mediated binding of NPM to the G4 prevents intramolecular contacts within the NPM IDR, promotes intermolecular NPM–RM interactions, and thus facilitates the LLPS. (**b**) Heterochromatin protein 1α (HP1α). The HR-mediated binding of HP1α to the G4 prevents HP1α HR–CTE contacts, stabilizes the extended HP1α conformation, promotes intermolecular HP1α–H3Kme2/3 conformation, and thus facilitates the LLPS and heterochromatinization.

SERBP1 assists in transcription, chromatin remodeling, and the assembly of promyelocytic leukemia (PML) bodies in the nucleus, while in the cytoplasm, it stabilizes mRNA and controls translation [55]. This protein was identified as a G4 interactor in the pull-down [48] and eCLIP [40] assays. It contains Arg-Gly/Arg-Gly-Gly repeat domains (RG/RGG), which are implicated in both the recognition of the G-rich sequences and LLPS [56]. Nucleophosmin, the multifunctional protein crucial for ribosome biogenesis [57], recognizes DNA G4s via its largely aromatic C-terminal domain and the neighboring IDR [58], which controls the propensity of this protein for homotypic versus heterotypic interactions [59]. The latter include IDR contacts with Arg-rich motifs of nucleolar proteins and supposedly contribute to the assembly of the nucleoli around rRNA [57] (Figure 2). HnRNP A1 recognizes G4s via its RGG domain. Together with the adjacent N-terminal domain UP1, comprising two RNA recognition motifs (RRMs), the RGG domain unfolds the G4s, and this helicase-like activity has been implicated in the telomere maintenance and transcription activation [51,52,60]. The LLPS of hnRNPA1, mediated by the C-terminal LCD, has not been described in the nucleus so far, while in the cytoplasm, it promotes the assembly of stress granules that may eventually maturate into pathogenic solid aggregates [61,62]. NCL, one of the essential nucleolar proteins, shares several functions and structural features with hnRNPA1 [63]. It contains four RRMs and an RGG-rich C-terminal IDR [64]. Both, the RRM and IDR of NCL are implicated in the G4 binding [65].

The FUS protein, one of the few TFs enriched at the endogenous DNA G4s and displaced efficiently by the G4-specific ligand PDS [66], also binds the G4s through the RGG domains. There are three such domains in FUS, and they adopt a so-called β-spiral structure stabilized by the adjacent Pro- and Arg-rich regions [53]. In addition to the G4 recognition and non-specific nucleic acid binding via electrostatic interactions, the RGG domains mediate the FUS self-association [67]. Both the RGG domains and the N-terminal LCD, rich in aromatic amino acids, are essential for the FUS separation [68,69], because RGG methylation [70] or LCD phosphorylation [71] abolish the FUS foci in the cell. As in the case of hnRNPA1, the LLPS and liquid-to-solid phase transitions of FUS are mostly associated with the pathological processes in the cytoplasm. The nuclear import restricts FUS transitions under normal conditions due to the chaperone-like activity of importin [72]. However, intranuclear FUS-driven LLPS has been observed on DNA damage and appears integral to the damage response initiation [73].

Additional examples of the known G4-binding LLPS drivers are provided in Table 1 along with a semi-quantitative evaluation of their relative LLPS capabilities using the RNAgranuleDB [74] and ParSe [75] tools. The former tool combines several sequence-based predictors that have been reviewed and verified extensively for RNA-binding proteins [76]. The latter tool is notable for distinguishing LLPS-prone and LLPS-incapable IDRs/LCDs. It relies on the assessment of the β turn propensity of an IDR/LCD based on the bulkiness of its “spacer” (supposedly inert) amino acid residues. Using the percentage of the LLPS-prone IDRs predicted using ParSe (see Table 1 footnote), we classified the G4 binders as robust (++), intermediate (+), and presumably weak (+/−) LLPS drivers. The RNAgranuleDB and ParSe data showed a reasonably weak correlation with each other, and future improvements in the algorithms are needed [77].

It can be concluded that LLPS drivers are abundant, if not enriched, in the G4 interactome. Nucleoplasm–cytosol shuttling RNA-binding LLPS drivers with a remarkable affinity for G4s typically contain Arg-rich domains that are capable of weak electrostatic/cation–pi interactions, and/or aromatic fragments that are capable of weak pi–pi interactions, which are commonly observed in cellular condensates [92,93] (Figure 1b). The direct effects of G4 DNA on the phase transitions of such RNA-binding proteins remain to be tested. One notable exception is SERBP1. The sensitivity of its separation to the G4 DNA was analyzed indirectly in a model system under biomimetic conditions [48]. Protocell-imitating giant membrane vesicles (GMVs) were loaded with G4-forming or non-G4 DNA. The former showed a reversible (temperature-dependent) clustering behavior consistent with the biomolecular condensate formation and enhanced uptake (likely due to trapping in the condensates). The subsequent pull-down experiments revealed SERBP1 as a major G4 binder in the GMVs. Both its interactions with the G4s and the LLPS were inhibited by the G4 ligand PDS. Although the biological relevance of the GMV model should not be overestimated, it provided the first direct evidence for the targetable G4 DNA-driven condensate formation in the native environment.

## 3. G4s Are Abundant in RNA Processing-Related Nuclear Condensates

**Figure 3:**
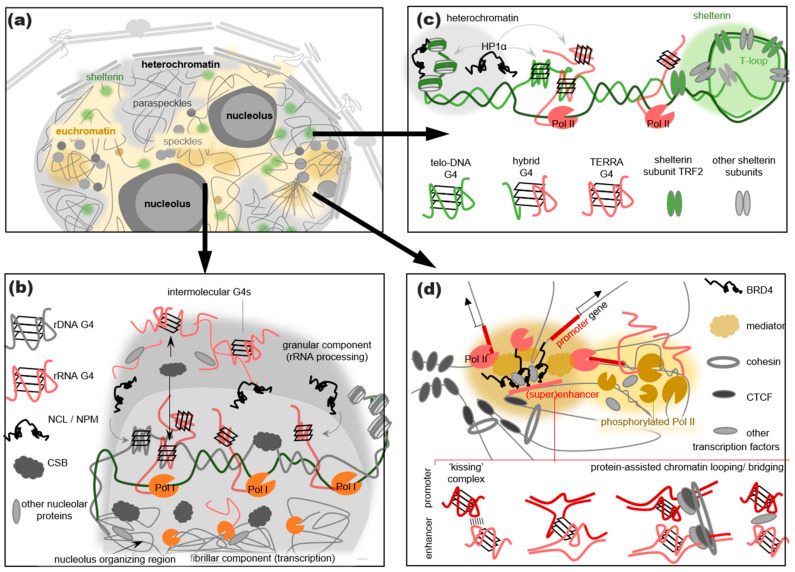
G4s in nuclear biomolecular condensates. (**a**) Schematic representation of typical nuclear condensates with G4-prone DNA/RNA. (**b**) Details on the nucleoli organization. (**c**) Details on the organization of sub-telomeric heterochromatin and shelterin. (**d**) Presumed organization of Pol II condensates upon active transcription.

Synthetic DNA G4s were typically used in interactome studies [34,35], GMV-based LLPS assays [48], and most other relevant in vitro experiments [82,94]. However in vivo, the primary role of the RNA G4s cannot be excluded. The abundant noncoding G4 motif-harboring transcripts appear to modulate the RNA processing-related nuclear bodies, such as speckles, paraspeckles and, perhaps, nucleoli [83,84,95] (Figure 3). Speckles are RNA protein granules enriched in pre-mRNA splicing factors (Ser-Arg-rich in particular) and assembled in the interchromatin regions [96]. Paraspeckles adjoin the speckles and are enriched in stress response or differentiation-related proteins and RNA [97]. Both paraspeckle-specific long noncoding nuclear enriched abundant transcript 1 (NEAT1) and speckle-specific noncoding nuclear enriched abundant transcript 2 (NEAT2, also known as MALAT1) contain several G4-prone sites [83,84]. These sites are crucial for the recruitment of an LLPS-driving transcription and splicing factor NONO [83,98], and the G4-disrupting mutations in MALAT1 alter splicing [99]. In addition to NONO, MALAT1 G4s have been shown to bind nucleophosmin and nucleolin [99] and may interact with other G4-recognizing RNA-binding LLPS drivers, such as FUS and hnRNP A1. However, these proteins are sporadically included in speckles/paraspeckles and appear non-essential for their assembly [96].

Nucleoli, the condensates in which rRNA is synthesized, are assembled around the so-called nucleolar organizer regions of genomic DNA that contain clusters of G4-harboring ribosomal gene repeats (rDNA) [100]. The transcription of rDNA by RNA Pol I requires the recruitment of Gly-Arg-rich (GAR) chromatin remodeling and a repair factor—cocaine syndrome protein B (CSB). This protein has recently been shown to recognize and resolve intermolecular rDNA G4s [94]. CSB is assumed to accumulate in R-loops and prevent excessive intermolecular G4 formation, which explains its importance for transcription maintenance in the nucleoli. The underlying mechanisms require further investigation. However, it appears safe to assume that the integral nucleoli components are accumulated at short-lived G4s. Apart from CSB, such G4s can recruit RGG- and IDR-containing presumed co-drivers of the LLPS-mediated nucleoli assembly NCL [101] and nucleophosmin [102], as well as the GAR domain-containing LLPS drivers CAR1 and fibrillarin [103]. The interactions between the G4s and CSB or NCL can be disrupted by G4 ligands, such as PDS, CX-5461, or CX-3543, leading to nucleoli dysfunction and abolished rRNA synthesis [94,104].

It should be emphasized once again that rDNA G4s are typically mentioned as possible targets of the ligands because most in vitro data were obtained using synthetic DNA. However, nucleoli staining using a G4-specific benzothiazole-based light-up probe thioflavin T (ThT) revealed a decreased foci number after the RNAse but not the DNAse treatment, pointing to the prevalence of RNA rather than DNA G4s [105]. In the bulk of the nucleus, the opposite situation was observed when staining with a distinct benzothiazole-based probe, IMT [106] (ThT only stains nucleoli). The RNAse treatment had little effect on the IMT foci, while the DNAse or urea treatment eliminated them, arguing for the overall prevalence of DNA rather than RNA G4s.

To summarize, the G4s in noncoding RNA and rDNA are capable of recruiting established and presumed LLPS drivers, presumably increasing their local concentrations to a critical point needed for a spontaneous phase transition. Since Arg-rich motifs (RM) are present in the majority of those proteins, G4s might contribute to LLPS through weak cation-π interactions (Figure 1c). In the nucleoli, the G4-induced conformational rearrangements of nucleophosmin favor its interactions with the RM-containing proteins (Figure 2). The formation of short-lived “bridging” intermolecular G4s may also contribute directly to the LLPS (Figure 3b). However, it remains to be clarified which particular structures (inter- or intramolecular DNA, RNA, or hybrid G4s in R-loops) play the key role.

## 4. G4s Promote the LLPS of Heterochromatin- and Shelterin-Assembling Proteins

The G4s appear to play context-dependent permissive/repressive roles in the gene expression, thus contributing to the cell-type-specific transcription control [9,13]. In particular, they have been detected in human heterochromatin [107] and may recruit gene-silencing chromatin remodelers—polycomb repressive complex 2 [35], linker histone H1 [82], and heterochromatin protein 1 isoform HP1α [91]. This does not necessarily contradict the acknowledged association between the G4s and active transcription [8]. Heterochromatinization might be initiated to prevent DNA damage upon excessive G4 formation [108], suggesting a negative feedback loop-like mechanism. A recent analysis of the murine genome using the transposase antibody fusion-based CUT&Tag technology [109] confirmed that the G4s existed irrespectively of the ongoing transcription [110]. The causal relationship between the G4s and the chromatin packaging will hopefully be further elucidated in future studies. Regardless, should H1 and HP1α accumulate at G4-rich sites, heterochromatinization likely occurs through their co-separation [82,111].

The in vitro assays with polynucleosomes evidenced that H1 is the key separation driver [112]. In the absence of competitors (linker histone analogs) or transcription-activating core histone modifications, H1 binds internucleosomal DNA at the nucleosome entry/exit points through electrostatic interactions, screens the DNA–DNA repulsion, and thus promotes the chromatin condensation. The condensation is often referred to as LLPS-mediated [113], since the highly disordered H1 and histone tails form transient contacts with each other and the DNA [114]. There is an ongoing dispute on whether rigidly packed nucleosome units can be regarded as liquid. However, in a silico demonstration of the nucleosome plasticity seems to have reconciled this problem [115]. The effects of G4s on the phase separation of polynucleosomes are yet to be clarified. The construction of a biologically relevant in vitro model is challenging but possible. Although G4s and nucleosomes are mutually exclusive, recent atomic force microscopy (AFM) studies provided indirect evidence for the G4 formation in linker (internucleosomal) DNA and the possible impact of G4s on the nearby nucleosome plasticity [116]. The studies in a simplified (histone-free) model revealed an enhanced H1-driven LLPS in the presence of parallel-stranded G4 DNA compared to the poly-A DNA [82]. This result was attributed to the increased multivalency and contact diversity. The B-DNA -> G4-DNA conformational transition, which exposed the hydrophobic DNA “facets” (outer G-tetrads) and facilitated π–π/cation–π interactions, whereas B-DNA was only accessible for the electrostatic interactions. Such an effect may be particularly pronounced in the case of parallel-stranded G4s because their propeller-type loops do not hinder access to the outer G-tetrads.

HP1α co-separates with H1 in vitro and plays an important accessory role in the heterochromatinization in vivo [112]. In solution, the dynamic equilibrium between the compact (autoinhibited) monomeric/dimeric and extended (oligomerization-prone) forms of HP1α is expected [117]. The compact forms are stabilized by the electrostatic interactions between the C-terminal extension (CTE) and the central unstructured “hinge” region (HR) or the N-terminal extension (NTE), and the dimers are held together through the contacts of the chromo-/chromoshadow domains (CD, CSD). The phosphorylation of the HP1α NTE and the HR-mediated binding of HP1α to the parallel-stranded G4s [91] shift the equilibrium toward the active form and eventually facilitates the LLPS, which has been demonstrated in vitro using single-molecule DNA curtain assays [117]. The presumed mechanism resembles the proposed model for nucleophosmin: the G4s assist in the LLPS by favoring intermolecular protein contacts over intramolecular ones (Figure 2). The preference of HP1α for the parallel-stranded G4s over the antiparallel ones or random sequences has been confirmed in vitro and in situ. The exogenous parallel-stranded G4s introduced into the cell nuclei outcompeted the genomic ones and disrupted the HP1α foci [91].

Importantly, the heterochromatin establishment at the sub-telomeric regions is dependent on the HP1α recruitment to the telomeric repeat-containing RNA (TERRA), rather than the DNA, and appears to be interlinked with the shelterin assembly [91,118] (Figure 3c). Shelterin also possesses characteristic features of the liquid condensates, which has been confirmed in cellulo using the optogenetic approach [119]. The LLPS-driving subunits of the shelterin complex are the telomeric repeat binding factors TRF1 and TRF2 [119]. They recognize telomeric sequences and reshape them into T-loops, ensuring ssDNA protection from nuclease hydrolysis. The IDRs and dimerization domains of TRF1/2 participate in multiple homo/heterotypic interactions, leading to the formation of condensates that control the telomerase access to DNA. TRF2 is enriched to some extent at G4-prone sites throughout the genome (e.g., in promoter regions) and can be outcompeted by the G4-binding ligands 360A [89] or PDS [120], suggesting a specificity for the G4 structures. The treatment using PDS was shown to disrupt shelterin. Nevertheless, the TRF2 interactions with telomeric DNA are likely sequence-dependent rather than secondary structure-dependent [114]. The in vitro studies of the triple (DNA–TRF2–TERRA) complexes evidenced that the TRF2 GAR domain bound the RNA G4s, while DNA was recognized by a distinct (DNA binding) domain in both the quadruplex and duplex forms [121]. Thus, it appears that shelterin scaffolding only requires G4 folding in TERRA [90].

## 5. G4s May Assist in Assembling Transcription Initiation- and Reparation-Related Condensates

A recent semi-comprehensive immunocytochemistry-based investigation of the G4 location relative to various nuclear condensates [122] revealed a significant colocalization with Pol II clusters and SC35 domains (nuclear speckles). The Pol II clusters were referred to as transcription factories—the immobile condensates were preassembled through the Pol II CTD-mediated LLPS, to which the DNA was supposedly recruited and through which it was supposedly reeled during the transcription elongation [123]. Such use of terminology could be misleading since the reported correlation between the Pol II clusters and speckles fits an alternative model [124] that highlights the role of enhancers/super enhancers (SEs) and does not require the Pol II immobilization. According to that alternative model, unphosphorylated Pol II and the multiple associated proteins, including the LLPS drivers from the bromodomain and extra terminal domain (BET) family, were initially recruited as enhancers/super enhancers and form so-called transcription initiation condensates. Following CTD phosphorylation, the elongation competent Pol II is released from those condensates to the proximal promoter, thus triggering transcription elongation. To enable the timely processing of the RNA obtained from the highly transcribed super-enhancer-regulated genes, the initial Pol II clusters were frequently associated with nuclear speckles [125].

The permissive role of the promoter/enhancer G4s in the gene expression can be attributed to the accumulation of TFs, including the LLPS drivers TAF15 [6], SP1 [78], and, possibly, BET proteins [79]. The direct binding to G4s in vitro was demonstrated for the BET protein BRD3 [34,79], which shares key structural features and functions with its homologues BRD4 and BRD2. These proteins recognize acetylated chromatin through their bromodomains (BD) and remodel nucleosomes to maintain Pol II processivity during the transcription elongation [126]. Their partially disordered extra terminal domains (ET) attract other TFs. For instance, BRD2 is notable for recruiting STAT3 to active enhancers [127], while BRD4 recruits the mediator complex, connecting SEs to Pol II [128]. The simultaneous recognition of the DNA structural features and acetylated histones through BDs may enhance the BET specificity for the promoter and enhancers, but conclusive evidence is lacking.

Another possibility is the transcription activation through the LLPS-driving bridging interactions [129]. The G4s may promote the DNA bridging interactions directly through intermolecular folding [12] or a loop-mediated “kissing complex” formation [130], as well as indirectly through recruiting chromatin looping-related zinc finger proteins (Figure 3d). Notable examples of such ZF proteins affine to G4s include Ying-Yang 1 [131], which mediates the formation of the enhancer–promoter contacts [132]; the CCCT-binding factor (CTCF) [11,133], which governs the cohesin positioning and insulates the topologically associating chromatin domains [134]; and the MYC-associated zinc finger protein MAZ [80], which works in concert with the CTCF to regulate the cohesin positioning [135].

All the above hypotheses imply that G4s can be prerequisites rather than mere consequences of chromatin opening upon active transcription [8]. The same applies to replication. Considering that G-rich motifs mark the replication origins [136], the pre-existing G4s may contribute to the recruitment and LLPS-mediated [137] assembly of the IDR-harboring origin recognition complex (ORC) for the subsequent ORC, Cdc6, and Cdt1-driven loading of the minichromosome maintenance helicases and replication initiations [138]. Although the ORC does not show a specificity for the DNA sequence, a secondary structure specificity cannot be excluded. Alternatively, the G4s might contribute to replication origin selection by maintaining a local low nucleosome density [117,139] without direct interactions with the ORC subunits.

Interestingly, despite the accumulation in the S phase, evident from both small-molecule-based [106] and antibody-based [4] imaging, G4s appear to implement their acknowledged regulatory functions, as well as presumed transcription initiation condensate-scaffolding and ORC-scaffolding functions in the G1 phase. During replication, they hamper fork progression and must be resolved timely by helicases to avoid DNA damage or histone code loss [140]. Treatment using G4-stabilizing small molecules or the disfunction of G4-unwinding helicases reportedly induces genetic instability due to the increased dsDNA break rates [7] or the epigenetic instability due to a pause-driven disbalance between the histone recirculation and recruitment [141]. However, under normal conditions, G4s show no significant correlation with the damage markers [122]. A peculiar case is an exposure to reactive oxygen species. Due to the low guanine redox potential, G4s take the brunt, becoming genomic oxidation hotspots [142,143]. At the same time, they appear to facilitate timely reparation by recruiting and activating the damage signaling and reparation factor poly(ADP-ribose) polymerase 1 (PARP1) [85,86].

The mechanisms of the G4 oxidation, base excision repair, and the possible consequences for transcription factor loading have been reviewed elsewhere [12]. The recent data hint at PARP1-mediated condensate remodeling at the damage sites [85]. First, PARP1 adds poly(ADP-ribose) chains (PAR) to itself (autoactivation) and the nearby proteins, which pauses replication or transcription due to the disruption of the respective condensates [144,145]. The PARylated proteins at the DNA damage sites recruit FUS [73] and prime it to the LLPS [146,147], thus scaffolding the reparation condensates. Once the reparation is complete, the PAR hydrolysis by PARG releases FUS, leading to the disassembly of the reparation condensates. This presumed mechanism has been verified in a model in vitro system and visualized using AFM [73].

## 6. Conclusions and Open Questions

In this review, we outlined the current understanding of the G4 contributions to the assembly and function of nuclear macromolecular condensates. This understanding is grounded by G4 interactome studies, in vitro LLPS assays, and intracellular imaging assays using antibodies for the G4s and condensate markers, as well as small-molecule light-up probes. The limitations of such studies should be taken into account.

First, short oligodeoxyribonucleotides prone to intramolecular folding were used as the model G4s in most of the in vitro experiments. However, the key role of the intermolecular structures, RNA G4s, or RNA/DNA hybrids in the nuclear condensates could not be excluded. Second, the in vitro LLPS assays rarely followed the complete protocols for a bona fide verification of the condensate liquid state. The intracellular LLPS verification was even more challenging, and the requirements were never fully met in the G4 studies. Classical immunofluorescence staining and the usage of light-up probes hardly provided sufficient spatiotemporal resolution, while optogenetic tools are not yet commonly accessible.

Despite the technical limitations, several lines of research have converged to support the importance of G4s in nuclear condensates. Some of the major results and interpretations are summarized below.

- G4s promote the LLPS of heterochromatin-associated proteins in artificial systems, but the biological relevance of these findings awaits verification.
- G4s promote the LLPS of RNA-binding proteins in the pseudo-cellular environment. These findings are in line with the studies of cytoplasmic condensates and may be relevant to the assembly of nuclear RNA processing factor-rich condensates, namely nucleoli, speckles, and paraspeckles.
- The integrity and/or functions of speckles/paraspeckles are disrupted by G4 mutations and G4-stabilizing ligands. The shelterin integrity and function are also disrupted by G4 ligands. The effects of these ligands are attributed to their interference with G4 protein interactions.
- The colocalization with Pol II clusters, TFs, and chromatin loop boundaries supports the idea that G4s assist in the transcription initiation. However, conclusive evidence is lacking. A comparison of transcription burst rates at G4-rich and non-G4 SEs could probably clarify this matter.

The proposed mechanisms for the G4-mediated LLPS can be classified as follows.

- The nucleobase exposure in the G4 outer tetrads and the adjoining ssDNA regions for transient π–π interactions with aromatic amino acid-rich proteins and cation–π interactions with Arg-rich ones (Figure 1).
- The exposure of a protein IDR/LCD for transient interactions with other macromolecules following a G4-binding-induced conformational transition (Figure 2).
- The accumulation of multiple IDR/LCD-containing proteins at the G4 repeats through the G4 recognition by the structured domains of these proteins or their partners (Figure 3b–d).
- The assembly of a transient nucleic acid “net” through the formation of G4–G4 kissing complexes, intermolecular G4 folding, or chromatin looping mediated by G4-binding proteins (Figure 3b–d).

Most G4-stabilizing small molecules are planar polyaromatic structures with positively charged (typically amino/guanidino group-harboring) substituents tailored to form stacking contacts with an outer G4 tetrad. They may interfere with transient π–π/cation–π interactions and outcompete the G4-binding proteins, including those involved in chromatin looping. This makes the typical G4 ligands prospective remodelers of the G4-containing nuclear condensates. The consequences of such a remodeling would be systemic at a cellular level and may outweigh the desired specific effects on G4 targets from oncogene promoters, telomeres, etc. This thesis undermines the predictability of G4 targeting with small molecules to some extent, but also points to exciting new possibilities for manipulating nuclear condensates.

## Figures and Tables

**Table 1:** Examples of G4-binding LLPS drivers.

Protein		G4 Binding (G4 Type)	LLPS Capability	
Code	Name		RNA Granule DB	ParSe *
SP1	Specificity protein 1	[78] (DNA)	1.1	++
FUS	Fused in sarcoma	[53,54] (DNA, RNA)	22	++
BRD3	Bromodomain-containing protein 3	[34,79] (DNA)	0.3	+/−
MAZ	MYC-associated zinc finger protein	[80] (DNA)	0.3	+
TAF15	TATA box-binding protein-associated factor 15	[6] (DNA)	15.7	++
hnRNPA1	Heterogeneous nuclear ribonucleoprotein A1	[51,81] (DNA, RNA)	18.8	+
H1	Histone H1	[82] (DNA)	6.4	−
NONO	Non-POU domain-containing octamer-binding protein	[83,84] (RNA)	10.4	+/−
PARP1	Poly [ADP-ribose] polymerase 1	[85,86] (DNA)	2.5	+/−
DDX1	DEAD-box helicase 1	[39,87] (DNA, RNA)	29.3	+/−
DDX5	DEAD-box helicase 5	[41] (DNA)	4.7	+/−
DDX24	DEAD-box helicase 24	[39] (DNA, RNA)	0.6	−
DBP1/2	D-box-binding PAR BZIP transcription factor	[42,44] (DNA, RNA)	1	+/−
NCL	Nucleolin	[50,88] (DNA, RNA)	3.8	+
NPM1	Nucleophosmin	[49] (DNA)	8.7	−
TRF2	Telomeric repeat-binding factor 2	[89,90] (DNA, RNA)	0.1	+/−
SERBP1	SERPINE1 MRNA binding protein 1	[48,56] (DNA, RNA)	15.4	+
HP1α	Heterochromatin protein 1, isoform α	[91] (DNA, RNA)	0.2	−

* ++, ≥40% pro-LLPS IDR; +, 20–40% pro-LLPS IDR; +/−, at least one pro-LLPS IDR.

## Data Availability

Not applicable.
